# The effects of N95 mask and face shield on speech perception among healthcare workers in the coronavirus disease 2019 pandemic scenario

**DOI:** 10.1017/S0022215120002108

**Published:** 2020-09-28

**Authors:** S V Bandaru, A M Augustine, A Lepcha, S Sebastian, M Gowri, A Philip, M D Mammen

**Affiliations:** 1 Department of Otorhinolaryngology, Christian Medical College, Vellore, India; 2 Department of Audiology, Christian Medical College, Vellore, India; 3 Department of Biostatistics, Christian Medical College, Vellore, India

**Keywords:** Masks, Audiometry, Speech, Personal Protective Equipment

## Abstract

**Objective:**

The current circumstances of the coronavirus disease 2019 pandemic necessitate the use of personal protective equipment in hospitals. N95 masks and face shields are being used as personal protective equipment to protect from aerosol-related spread of infection. Personal protective equipment, however, hampers communication. This study aimed to assess the effect of using an N95 mask and face shield on speech perception among healthcare workers with normal hearing.

**Methods:**

Twenty healthcare workers were recruited for the study. Pure tone audiometry was conducted to ensure normal hearing. Speech reception threshold and speech discrimination score were obtained, first without using personal protective equipment and then repeated with the audiologist wearing an N95 mask and face shield.

**Results:**

A statistically significant increase in speech reception threshold (mean of 12.4 dB) and decrease in speech discrimination score (mean of 7 per cent) was found while using the personal protective equipment.

**Conclusion:**

Use of personal protective equipment significantly impairs speech perception. Alternate communication strategies should be developed for effective communication.

## Introduction

Efficient communication is the key to effective healthcare. The current circumstances of the coronavirus disease 2019 (Covid-19) pandemic have necessitated the routine use of personal protective equipment (PPE) in all areas of hospitals, from out-patient clinics to operating theatres. An N95 mask and face shield are being used as PPE to protect from aerosol-related spread of infection.

With increased workload, effective communication between healthcare workers and between healthcare workers and patient is essential to ensure that healthcare is delivered effectively. The use of PPE, however, greatly hampers communication. The visual cues from lip reading are completely cut off and views of facial expressions are diminished greatly. Patients may not completely understand the doctors’ instructions. Considering the ambient noise, communication between healthcare workers may require multiple repetition and increased strain on listening. Further, communication errors are likely, with the potential for grave consequences. In the operating theatre or during procedures, assisting staff may not reliably follow the instructions of the operating surgeon.

In our tertiary care ENT out-patient set up, healthcare workers are now required to routinely wear an N95 mask and face shield in order to limit infection, and this level of PPE seems to be the minimum requirement in operation theatres as well. Our aim was to quantitatively assess the effect of using an N95 mask and face shield on speech understanding among healthcare workers with normal hearing by determining its effect on speech reception thresholds and speech discrimination scores.

## Materials and methods

This prospective observational study was conducted in the out-patient ENT clinic of our tertiary care referral centre in south India, after institutional review board and ethical committee clearance. Healthcare workers with normal hearing, in the age group of 20–60 years, working in the ENT out-patient clinic, were recruited for the study. They underwent pure tone audiometry to ensure normal hearing, defined as a pure tone average of less than 25 dB at 500, 1000 and 2000 Hz. Those with external or middle-ear pathology detected on otoscopy were excluded. Healthcare workers aged over 60 years were excluded, to negate the effect of presbycusis.

After obtaining informed consent and performing otoscopy, the participants underwent routine pure tone audiometry using a Grason-Stadler GSI-61^™^ clinical two-channel audiometer. Pure tone air conduction and bone conduction thresholds were obtained for frequencies of 250–4000 Hz. Thresholds up to 25 dB across the frequencies 250–2000 Hz were considered normal. The pure tone average was calculated using thresholds at 500, 1000 and 2000 Hz, and was also used to check the validity of the speech audiometry results. This was achieved by checking there was not more than 12 dB discrepancy between the pure tone thresholds and the speech reception threshold.

**Fig. 1.**
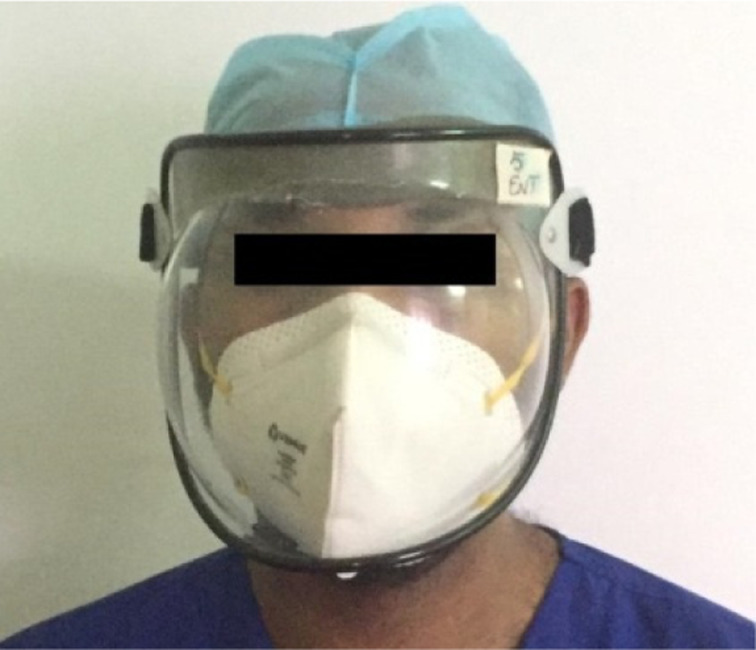
The N95 mask and face shield used by the audiologist during speech audiometry testing with personal protective equipment.

Once a normal hearing threshold was ascertained, the volunteers were subjected to speech audiometry to determine speech reception threshold and speech discrimination score. Speech audiometry was then repeated with the audiologist using an N95 mask (Venus V-44 respirator N95 mask; Venus Safety & Health, Navi Mumbai, India) and face shield (polycarbonate), as shown in Figure 1. The speech stimuli were presented through the audiometer to each ear separately using a headphone.

The speech reception threshold was estimated using a validated list of 50 spondee words recommended by the American Speech–Language–Hearing Association.^1^ These are two-syllable words that have equal stress on both syllables (e.g. ‘tooth brush’, ‘play ball’, ‘birthday’). A volume unit meter was used to obtain equal syllabic stress. The words were initially presented 20 dB above the pure tone audiometry threshold. If the response was correct, intensity was decreased by 10 dB steps, until the subject stopped responding. If the subject responded incorrectly, intensity was increased in 5 dB steps. The speech reception threshold was determined as the lowest hearing level (intensity) at which the subject could correctly recognise (perceive and repeat) the speech stimuli 50 per cent of the time.^2^

An open set of monosyllabic phonetically balanced words was used to determine the speech discrimination score: the subject repeated the words, with no choice of options. Standard word lists for determining the speech discrimination score included those issued by the Psycho-Acoustic Laboratory and the Central Institute for the Deaf W-22 word list for auditory testing.^3,4^ To suit the Indian population, our study employed a validated list of phonetically balanced words, adapted from Psycho-Acoustic Laboratory and Central Institute for the Deaf W-22 lists. The words were presented at a level 40 dB above the speech reception threshold. A list of 20 words was presented and the number of correct responses was expressed as a percentage. The speech reception threshold and speech discrimination score were then measured in the other ear in a similar manner, using a different set of spondee words and phonetically balanced words.

Speech audiometry (speech reception threshold and speech discrimination score measurement) was then repeated with the audiologist wearing an N95 mask and face shield. The new speech reception threshold was calculated while the audiologist was using the PPE. The speech discrimination score was calculated again, with the PPE, by presenting the stimuli 40 dB above the initial speech reception threshold calculated without PPE. This was done to estimate the degree of hearing difficulty faced by the subject when the examiner spoke normally (and not in a louder tone) even while using PPE. Hence, this simulates the healthcare ground situation where one tends to speak in a natural tone while wearing a mask, or more softly than normal, because of the positive feedback obtained with the occlusion effect of the mask. The speech reception threshold and speech discrimination score while using the PPE were compared to the initial measurements obtained when PPE was not used.

A pilot study was conducted on five volunteers. The required sample size was calculated based on the results obtained. For a power of 90 per cent and 5 per cent error, the minimum sample size required was 11 subjects. In order to explore additional comparisons, 20 participants were recruited for the study. Data were summarised using mean and standard deviation values for continuous variables, and frequency and percentage values for categorical variables. The pre–post changes were analysed using a paired *t*-test. Independent *t*-tests were used to compare the pre-, post- and change in speech reception threshold and speech discrimination score for the categorical demographic variables. For all comparisons, the level of significance was set at 5 per cent. Analysis was performed using Stata®/IC16.0 statistical software.

## Results

Twenty healthcare workers (10 men and 10 women) at the ENT out-patient clinic who volunteered for the study were recruited. Both ears were tested separately for each volunteer and therefore a total of 40 ears were studied. Our youngest subject was 23 years old, while the oldest was 54 years old (mean age of 40 years). There were 15 doctors, 3 nurses and 2 medical records officers in the study population.

**Fig. 2.**
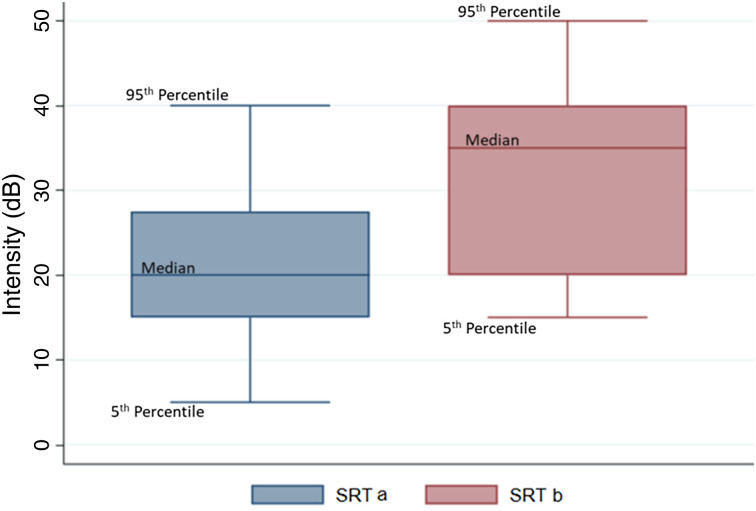
Box plot indicating the distribution of speech reception threshold intensity. SRT a = speech reception threshold without using personal protective equipment; SRT b = speech reception threshold while using personal protective equipment

The speech reception threshold ranged from 5 dB to 40 dB before using the PPE. The thresholds increased while using the PPE, ranging from 15 dB to 50 dB, as shown in Figure 2. A mean increase of 12.4 dB was observed.

**Fig. 3.**
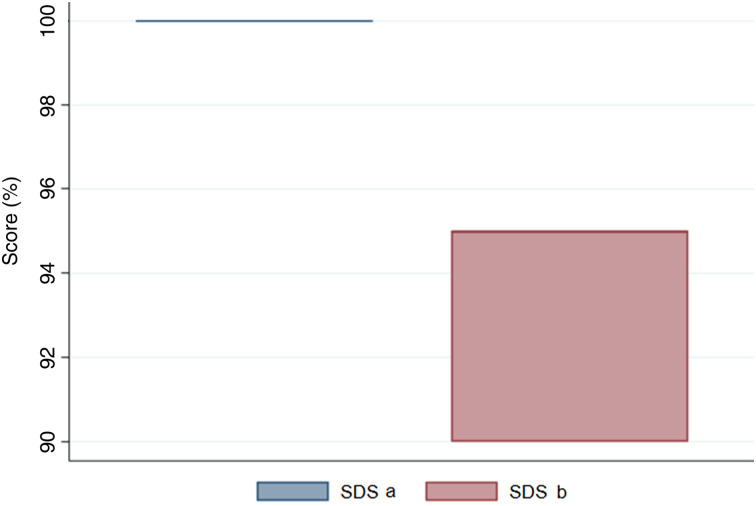
Box plot indicating the distribution of speech discrimination scores. SDS a = speech discrimination score without using personal protective equipment; SDS b = speech discrimination score while using personal protective equipment

The speech discrimination score was 100 per cent for all the participants before using the PPE. It decreased while using the PPE, ranging from 90 per cent to 95 per cent, as shown in Figure 3. A mean decrease of 7 per cent was observed.

Table 1 summarises the mean values of the speech reception threshold and speech discrimination score obtained with and without using PPE, for the 40 ears. There was a statistically significant increase in speech reception threshold and a decrease in speech discrimination scores with the use of PPE; the *p*-values obtained for both parameters were less than 0.0001 on paired *t*-test.

**Table 1.** Speech reception thresholds and speech discrimination scores with and without PPE use

Parameter	Ears (*n*)	With or without PPE	Mean value	SD	*P*-value
SRT (dB)	40	Without PPE	20.375	8.9	<0.0001
		With PPE	32.75	10.4	
SDS (%)	40	Without PPE	100	0	<0.0001
		With PPE	93.2	2.4	

PPE = personal protective equipment; SD = standard deviation; SRT = speech reception threshold; SDS = speech discrimination score

The changes in speech reception threshold and speech discrimination score measurements were further analysed with respect to age, gender and occupation. The results are summarised in Table 2. There were no statistically significant differences in the changes in speech reception threshold and speech discrimination score values obtained with and without using PPE when comparing between different age groups (20–40 years *vs* 41–60 years), sex (female and male) and occupation (doctors *vs* nurses and medical records officers).

**Table 2.** Comparison of changes in speech reception thresholds and speech discrimination scores according to age, sex and occupation

Parameter	Ears (*n*)	Change in SRT (mean (SD); dB)	*P*-value	Change in SDS (mean (SD); %)	*P-*value
Age					
– 20–40 years	20	11.8 (5.9)	0.08	6.2 (2.1)	0.43
– 41–60 years	20	13.0 (3.8)		7.5 (2.6)	
Sex					
– Male	20	12.8 (5.3)	0.64	6.8 (2.5)	0.85
– Female	20	12.0 (4.7)		6.9 (2.4)	
Occupation					
– Doctors	30	11.8 (5.3)	0.23	6.6 (2.3)	0.31
– Others	10	14.0 (3.1)		7.5 (2.6)	

SRT = speech reception threshold; SD = standard deviation; SDS = speech discrimination score

## Discussion

The importance of communication in all realms of human interaction is well understood. In the healthcare setup, effective communication among healthcare workers, and between healthcare workers and the patient or patient's caregivers, is key to the effective delivery of healthcare. Most healthcare settings are usually overcrowded, especially those in developing countries which cater to large numbers of patients with limited infrastructure. Aside from the resulting ambient noise, healthcare staff are likely to be working under significant work pressures. The Covid-19 pandemic has put additional burden on these already strained healthcare systems and personnel.

Given the risk of aerosol-related spread of infection, all levels of healthcare workers are required to use additional PPE at work. In the ENT out-patient setting at our tertiary care centre, the risk of aerosol generation has necessitated the use of an N95 mask and a face shield while interacting with patients. The operating theatres have also witnessed an increased use of PPE, because of the risk of aerosol generation during intubation and most ENT procedures. With the required PPE on, it is a common experience to have to repeat oneself multiple times to convey information to others in the healthcare team or to the patient. It was also felt that there was frequent miscommunication between healthcare workers, which could lead to potential medical errors.

This study attempted to quantitatively assess the effect of using PPE (N95 mask and face shield) on communication. Speech audiometry tests comprise both the audibility component (loss of sensitivity) and the distortion component (loss of clarity), assessed through measurement of the speech reception threshold and speech discrimination score respectively. The results of our study clearly demonstrate a significant increase in the speech reception threshold (mean of 12.4 dB) with the use of an N95 mask and a face shield. This result is comparable to a previous study on the degradation of speech reception associated with the use of medical masks, which recorded an attenuation of about 12 dB with the N95 mask.^5^

The speech discrimination score showed a worsening of about 7 per cent when the stimuli were presented at the same level with PPE versus without PPE. The presentation level was kept as 40 dB above the speech reception threshold obtained when not using PPE, because one tends to speak at a natural tone despite using PPE. The occlusion effect of the face mask tends to produce a positive feedback effect of speech loudness, which may in fact cause one to speak with a softer tone than normal. This positive feedback effect was not however accounted for in our study, as the phonetically balanced word list was delivered through an audiometer at a set level of 40 dB above the speech reception threshold obtained without using PPE. Although a statistically significant difference is demonstrated in the speech discrimination score values without PPE versus with PPE, the difference may well be larger in the regular setting.

Our study was performed in a sound-treated audiology setting in order to standardise the environment for quantitative assessment. However, most conversations in the healthcare setting occur in the scenario of significant ambient noise. This may further impair speech perception and intelligibility. In a study by Mendel *et al*. using surgical masks, there was a significant difference in the spectral analysis of speech stimuli with and without the mask. They did not find any difference in speech understanding between normal hearing and hearing-impaired individuals while using a surgical mask, but the presence of background noise (dental office noise) decreased speech understanding in both groups.^6^ Ideally, estimation should be conducted in the out-patient clinic setting; however, it is difficult to ensure a standard ambient noise and presentation level, to obtain reliable results. Hence, testing was carried out in a sound-treated room in our study.

The role of cues obtained from lip reading and facial expressions in the perception of speech cannot be ignored. These might have a negligible role in a normal hearing individual and in a quiet environment, but not for those with hearing impairment and in the presence of background noise. Atcherson *et al*., in their study on speech perception in noise when using surgical masks and transparent masks, found that while normal hearing individuals did not require visual cues, hearing-impaired individuals did better when a transparent mask was used.^7^ The stress and psychological effect of being in an unfamiliar environment, as for a patient in the hospital, can also impair speech understanding.^8^

In our study, age, gender and occupation had no statistically significant correlations with changes in speech reception threshold and speech discrimination scores, suggesting that this impairment in communication while using PPE is applicable to all healthcare workers. The impairment in speech perception while using PPE was evident despite participants being tested in ideal conditions and with the possibility of familiarisation of words associated with repeated testing.

- N95 masks and face shields are being used to protect from aerosol-related spread of infection
- However, this personal protective equipment (PPE) hampers communication
- This study found a significant increase in speech reception threshold (mean of 12.4 dB) with PPE use
- The speech discrimination score worsened by 7 per cent with PPE (*vs* without PPE) when stimuli were presented at the same level

Although a few previous studies have estimated the impairment in speech perception associated with face mask use, to our knowledge this is the first study to quantify the effect of using an N95 mask and face shield (as warranted by the current Covid-19 pandemic), on speech perception. Further studies on the compounded effect of various environmental variables on speech perception while using PPE will help to qualify these results substantially. The findings of this study justify working towards making the healthcare environment more conducive for effective communication, both among healthcare workers and between healthcare workers and the patients or their caregivers. The use of extra signage in the healthcare setting, adequate lighting, sign language for common instructions, and patient information hand-outs on disease conditions or hand-outs giving instructions may help overcome this communication barrier.

## Conclusion

While PPE has become an indispensable part of healthcare, its use significantly hampers communication, as evidenced by increased speech reception thresholds and decreasing speech intelligibility. It is important for healthcare workers to be conscious of this when communicating with each other and with the patient or their caregivers, to avoid errors and ensure effective delivery of healthcare. Alternative communication strategies may also be explored where appropriate to ensure effective communication.
